# Potential treatment of squamous cell carcinoma by targeting heparin-binding protein 17/fibroblast growth factor-binding protein 1 with vitamin D_3_ or eldecalcitol

**DOI:** 10.1007/s11626-024-00913-3

**Published:** 2024-05-07

**Authors:** Tomoaki Shintani, Mirai Higaki, Siti Nur Zawani Rosli, Tetsuji Okamoto

**Affiliations:** 1 Center of Oral Clinical Examination, Hiroshima University Hospital, Hiroshima, 734-8551 Japan; 2 Department of Molecular Oral Medicine and Maxilofacial Surgery, Graduate School of Biomedical and Health Sciences, Hiroshima University, Hiroshima, 734-8553 Japan; 3 Infectious Disease Research Center, Institute for Medical Research, Bacteriology Unit, National Institutes of Health, Ministry of Health Malaysia, 40170 Setia Alam, Malaysia; 4 School of Medical Sciences, University of East Asia, Shimonoseki, 751-8503 Japan

**Keywords:** Fibroblast growth factor, Squamous cell carcinoma (SCC), Heparin-binding protein17, Fibroblast growth factor-binding protein 1, Vitamin D3

## Abstract

Heparin-binding protein 17 (HBp17), first purified in 1991 from the conditioned medium of the human A431 squamous cell carcinoma (SCC) cell line, was later renamed fibroblast growth factor-binding protein 1 (FGFBP-1). HBp17/FGFBP-1 is specifically expressed and secreted by epithelial cells, and it reversibly binds to fibroblast growth factor (FGF)-1 and FGF-2, as well as FGFs-7, -10, and -22, indicating a crucial involvement in the transportation and function of these FGFs. Our laboratory has investigated and reported several studies to elucidate the function of HBp17/FGFBP-1 in SCC cells and its potential as a molecular therapeutic target. HBp17/FGFBP-1 transgene exoression in A431-4 cells, a clonal subline of A431 that lacks tumorigenicity and does not express HBp17/FGFBP-1, demonstrated a significantly enhanced proliferation in vitro compared with A431-4 cells, and it acquired tumorigenicity in the subcutis of nude mice. Knockout (KO) of the HBp17/FGFBP-1 by genome editing significantly suppressed tumor growth, cell motility, and tumorigenicity compared with control cells. A comprehensive analysis of expressed molecules in both cell types revealed that molecules that promote epithelial cell differentiation were highly expressed in HBp17/FGFBP-1 KO cells. Additionally, we reported that 1α,25(OH)_2_D_3_ or eldecalcitol (ED-71), which is an analog of 1α,25(OH)_2_D_3_, suppresses HBp17/FGFBP-1 expression and tumor growth in vitro and in vivo by inhibiting the nuclear factor kappa-light-chain-enhancer of activated B cells signaling pathway. Here, we discuss the prospects of molecular targeted therapy targeting HBp17/FGFBP-1 with 1α,25(OH)_2_D_3_ or ED71 in SCC and oral SCC.

## Introduction

Fibroblast growth factors (FGFs), of which 23 family members have been identified to date, are cell signaling proteins that were originally isolated from the pituitary gland and hypothalamus as growth factors that promoted fibroblast proliferation (Gospodarowicz *et al*. 1974; Burgess and Maciag 1989; Klagsbrun 1989; Itoh and Ornitz 2011). FGFs play an important role in tumor growth and invasion by inducing angiogenesis as well as promoting tumor cell proliferation in squamous cell carcinoma (SCC), salivary gland cancer, hepatocellular carcinoma, and malignant melanoma (Herlyn *et al*. 1987; Albino *et al*. 1991; Myoken *et al*. 1994; 1995; Rols *et al*. 1998). FGF-1 and FGF-2 are secreted although they have no signal sequence for secretion (Gospodarowicz *et al*. 1974; Abraham *et al*. 1986; Wang *et al*. 1989; Schulze-Osthoff *et al*. 1990; Burland *et al*. 1992; Bennett *et al*. 1995). However, the secretory mechanism of these prototypical FGFs remains unknown (Soutter *et al*. 1993; Seno *et al*. 1998).

In 1991, Wu *et al*. (1991) discovered and reported heparin-binding protein 17 (HBp17), a 17-kDa heparin-affinity-secreted protein with a signal sequence. HBp17, which originally copurified with FGF-2 from medium conditioned by A431 human epidermoid carcinoma cells, was shown to bind both FGF-1 and FGF-2 in a noncovalent and reversible manner, and based on this property it was predicted to regulate the extracellular availability and biological activity of FGFs (Wu *et al*. 1991; Yoshimura *et al*. 1998; Harris *et al*. 2000; Lametsch *et al*. 2000). HBp17 was later renamed fibroblast growth factor-binding protein 1 (FGFBP-1), and we refer to this protein as HBp17/FGFBP-1. Czubayko *et al*. (1997) showed that the depletion of endogenous HBp17/FGFBP-1 in cancer cells by targeting specific ribozymes reduced the growth and angiogenesis of xenograft tumors in mice. This suggested that HBp17/FGFBP-1 promotes angiogenesis in human tumors (Folkman and Hanahan 1991; Czubayko *et al*. 1997).

Many recent studies have revealed that vitamin D_3_ has diverse biological actions, which influence anticancer intracellular mechanisms and carcinogenesis, and vitamin D supplementation has various preventive effects (Starska-Kowarska 2023). The suppression of key intracellular signaling pathways, nuclear factor kappa-light-chain-enhancer of activated B cells (NF-κB), protein kinase B (AKT), mitogen-activated protein kinase/extracellular signal-regulated kinase, and class I phosphoinositide 3-kinase/AKT, or the inhibition of the cell cycle mediates the activities of vitamin D_3_ (Bikle 2021; Colotta *et al*. 2017). These pathways also inhibit proliferation and angiogenesis and appear to reduce the efficacy of head and neck cancer (HNC) chemotherapy (Koll *et al*. 2023). Additionally, vitamin D_3_ and its derivatives may act as antioxidants, anti-inflammatory agents, immunoprotectants, immunomodulators, regulators of cellular oncogenic signaling and apoptosis, and cell cycle and angiogenesis regulators (Chen *et al*. 2013; Singh *et al*. 2017). Proteomic profiling studies have confirmed that vitamin D_3_ negatively regulates carcinogenic transcription, stimulates many key genes encoding antioxidant enzymes, and promotes significant changes in other intracellular proteins via microRNA activity, a phenomenon essential for HNC carcinogenesis (Starska-Kowarska 2023). Recent studies revealed a quantitative association between vitamin D exposure and the incidence of HNC (Starska-Kowarska 2023). Additionally, the preventive effects of vitamin D in precancerous lesions of the head and neck and its role as a predictor of mortality, survival, and recurrence of HNC have been widely discussed (Mäkitie *et al*. 2020; Pu *et al*. 2021). This review will discuss the effect of vitamin D_3_ on the expression of HBp17/FGFBP-1 in oral squamous cell carcinoma (OSCC) and whether it can be further related to the prevention and treatment for OSCC.

### Expression of HBp17/FGFBP-1 in cultured cells

Wu *et al*. (1991) reported that HBp17/FGFBP-1 mRNA was expressed in normal keratinocytes and SCC cells, but not in normal human fibroblasts, fetal liver cells, hepatocellular carcinoma cells, or breast cancer cells. Sarcoma cells, melanoma cells, and most adenocarcinoma cells were also negative (Wu *et al*. 1991). The A431-4 nontumorigenic variant of A431 cell (Buss *et al*. 1982) did not express detectable HBp17/FGFBP-1 mRNA levels (Wu *et al*. 1991). Furthermore, Okamoto *et al*. (1996a, b) reported that normal epithelial cells derived from the salivary gland and cells derived from pleomorphic adenoma (Okamoto *et al*. 1996a), a benign salivary gland tumor, also expressed HBp17/FGFBP-1 mRNA. These results revealed that HBp17/FGFBP-1 expression is preferentially observed in squamous epithelial cells (Wu *et al*. 1991). Additionally, they suggested HBp17/FGFBP-1 is involved not only in tumorigenesis but also in normal functions in epithelial tissues. Subsequently, Beer *et al*. (2005) demonstrated that HBp17/FGFBP-1 also bound FGFs -7, -10, and -22, and provided evidence that it may play a role in epithelial wound repair (Beer *et al*. 2005).

### Acquisition of the tumorigenic potential of HBp17/FGFBP-1-transfected A431-4 cells in nude mice

A431 cells and the clonal derivative A431-4 both expressed FGF-1 and FGF-2, but only A431-4 cells did not express HBp17/FGFBP-1 (Wu *et al*. 1991). HBp17/FGFBP-1-transfected A431-4 cells produced palpable tumors in nude mice in 6–8 wk, but A431-4 cells that were transfected with empty vector did not form tumors even after 14 wk (Liu *et al*. 2002). Secondary and tertiary tumors derived from HBp17/FGFBP-1 transfected A431-4 cells were increasingly more tumorigenic than the original HBp17/FGFBP-1 cDNA transfectants. The tumorigenicity of the A431-4 transfectants correlated with HBp17/FGFBP-1 mRNA expression levels, but not with the FGF-1 or FGF-2 levels, as determined by northern blotting. These results indicated that HBp17/FGFBP-1 plays a role in the tumorigenesis of SCC through its interactions with FGF-1 and FGF-2. FGF accumulation in the culture supernatant is attributed to the ability of HBp17/FGFBP-1 to release FGFs from the extracellular matrix (ECM) (Liu *et al*. 2002). FGF-2 can be dissociated from ECM by treatment with high salt and heparinase-like enzymes (Baird and Ling 1987). Heparan sulfate proteoglycans, which are constituent molecules of the ECM, such as perlecan, serve as reservoirs for FGFs (Mongiat *et al*. 2001). Thus, the release of FGFs by HBp17/FGFBP-1 likely facilitates the autocrine or paracrine actions of FGFs.

### HBp17/FGFBP-1 gene knockout in SCC and OSCC

HBp17/FGFBP-1-knockout (KO)-SCC or OSCC cells were isolated and developed using clusters of regularly interspaced short palindromic repeats (CRISPR) and CRISPR-associated protein 9 (CRISPR/Cas9) genome editing technology. The amount of FGF-2 secreted into the culture supernatant decreased in HBp17/FGFBP-1-KO cells compared with that in wild-type (WT) cells (Shintani *et al*. 2021). Functional analysis revealed that proliferation, colony formation, and cell motility in vitro were inhibited in HBp17/FGFBP-1-KO cells. Tumor formation was not observed in mice transplanted with one of the two clones of HBp17/FGFBP-1-KO A431 cells in vivo, which confirmed the significant difference in growth in vitro between HBp17/FGFBP-1-KO and WT cells, indicating that HBp17/FGFBP-1 is potentially a potent therapeutic target in SCC/OSCC cells. Microarray and proteomic analyses revealed that knockout of HBp17/FGFBP-1 in A431 cells induced the expression of cornified envelope-associated mRNAs and proteins, which are terminal differentiation markers of squamous epithelial cells, compared to A431 WT cells (Table 1). Upregulation of fatty acid-binding protein 5 (FABP5), small proline-rich protein 1A (SPRR1A), small proline-rich protein 1B (SPRR1B), Involucrin (IVL), Loricrin (LOR), and Filaggrin (FLG) mRNAs in HBp17/FGFBP-1-KO A431 cells was verified by qRT-PCR. Furthermore, the protein expression of FABP5, SPRR1A, SPRR1B, and IVL in A431-HBp17-KO cells was also revealed to be upregulated compared to A431 WT cells by western blotting and immunohistochemial analysis, supporting the mRNA quantification results. Thus, we showed that HBp17/FGFBP-1 has a novel function in regulating SCC/OSCC cell differentiation (Shintani *et al*. 2021).

**Table 1.** The fold change of the top 10 genes and proteins which were upregulated in HBp17/FGFBP-1 knockout cells compaired with control

Gene	Fold change	Protein	Fold change
FABP5	179.61	AKR1C3	8.37
S100A9	54.67	KRT1	8.28
S100A8	40.90	AKR1C2	6.22
SPRR1A	33.52	CA2	5.04
AKR1C3	26.10	FABP5	4.92
AKR1C2	12.50	SLPI	3.71
SPRR1B	7.00	SERPINB3	3.50
SPRR2D	4.53	ATP2B4	3.34
SLPI	4.39	S100A8	3.04
KRT1	3.94	S100A9	2.95

*FABP5* fatty acid-binding protein 5, *S100A9* Protein S100-A9, *S100A8* Protein S100-A8, *SPRR1A* Cornifin-A, *AKR1C3* Aldo–keto reductase family 1 member C3, *AKR1C2* Aldo–keto reductase family 1 member C2, *SPRR1B* Cornifin-B, *SPRR2D* small proline-rich protein 2D, *SLPI* antileukoproteinase, *KRT1* keratin, type II cytoskeletal 1, *CA2* carbonic anhydrase II, *SERPINB3* serpin peptidase inhibitor, clade B (ovalbumin), member 3, *ATP2B4* ATPase plasma membrane Ca2 + transporting 4

### The potential of HBp17/FGFBP-1 as a diagnostic or prognostic biomarker

HBp17/FGFBP-1 inhibition suppressed the growth of tumor xenografts and angiogenesis in mice (Czubayko *et al*. 1997). We examined the expression of HBp17/FGFBP-1, FGF-2, and VEGF-A in surgically resected human tissues, including normal mucosa, hyperplasia, dysplasia of different degrees, and oral OSCC, by immunohistochemical analysis, and revealed that HBp17/ FGFBP-1, FGF-2, and VEGF-A expressions also increased with the severity of epithelial dysplasia, and their expression scores were highly correlated at all stages of the multistage development of SCC (Begum *et al*. 2007). In addition, we found significant associations between microvessel density (MVD) and HBp17/FGFBP-1, FGF-2, and VEGF-A expressions (Begum *et al*. 2007). An interesting result of the study was the significant increase in MVD observed in severe rather than moderate dysplasia and OSCC. The decreased MVD in OSCC compared with severe dysplasia may be due to a decreased need for further angiogenesis (Li *et al*. 2005).

### HBp17/FGFBP-1 expression was downregulated by 1α,25(OH)_2_D_3_ or eldecalcitol (ED-71) via NF-κB signaling pathway

We tested the hypothesis that HBp17/FGFBP-1 expression was regulated by NF-κB by manipulating this particular binding site because of the existence of NF-κB site in the HBp17/FGFBP-1 promoter region (Harris *et al*. 1998). We investigated the effect of VD_3_ on the expression of HBp17/FGFBP-1 in HO-1-u-1 (UE) OSCC cells. HBp17/FGFBP-1 mRNA expression was downregulated in UE cells treated with VD_3_. Additionally, NF-κB activity was downregulated because of IκBα upregulation in those cells (Rosli *et al*. 2013). Moreover, the FGF-2 concentration of the culture medium in UE cells treated with VD_3_ was reduced compared with the control (Rosli *et al*. 2014). In fields other than cancer research, recent studies have shown that 1a,25(OH)_2_D_3_ (VD_3_), which has potent anti-inflammatory properties, can reduce intestinal inflammation by reducing NF-κB activation (Yang *et al*. 2022; Munem *et al*. 2023).

**Figure 1.**
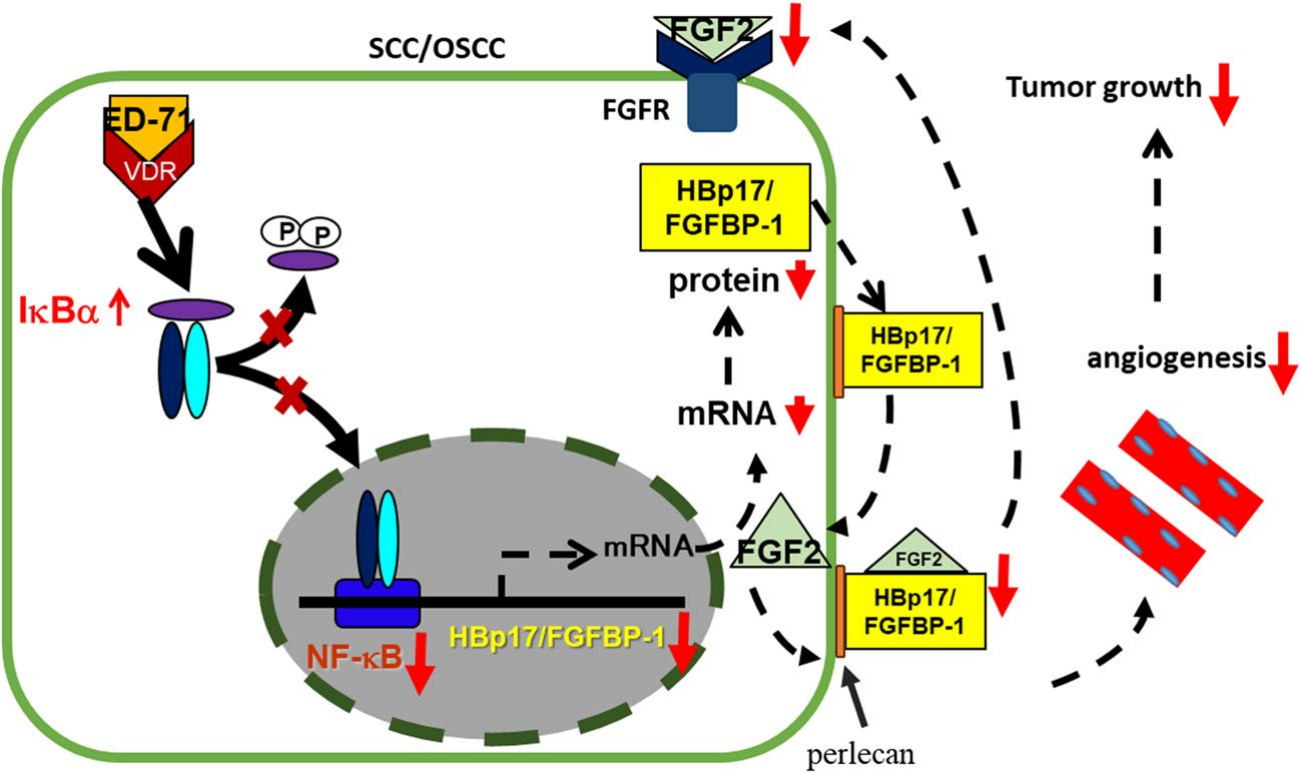
Summary of HBp17/FGFBP-1 expression suppression and tumor growth suppression by ED-71 or 1α,25(OH)_2_D_3_ (VD_3_) in SCC/OSCC cells. ED-71 or VD_3_ suppressed HBp17/FGFBP-1 expression by upregulating IκBα expression in the NFκB signaling pathway after binding to the VDR and suppressing NFκB signaling in SCC/OSCC cells.

We next examined the potential antitumor effect on SCC/OSCC cells in vitro and in vivo of eldecalcitol (ED-71), an analog of VD_3_, which is approved in Japan for use as an antiosteoporosis medication and has a longer half-life in vivo (Shintani *et al*. 2016, 2017). ED-71 has one-third to one-eighth of the activity of calcitriol in stimulating target genes due to its low affinity for vitamin D receptor (Saito and Harada 2014). However, its high affinity for vitamin D-binding protein, which has a longer half-life than other analogs and calcitriol, compensated for this low affinity (Hatakeyama *et al*. 2007). The in vitro growth assay revealed that ED-71 dose-dependently inhibited the growth of SCC/OSCC cell lines. Furthermore, NF-κB signaling pathway inhibition by ED-71, as with VD_3_, suppressed the HBp17/FGFBP-1 expression. A luciferase reporter assay was used to suppress HBp17/FGFBP-1 promoter activity within the − 217 and + 61 regions (putative NF-κB binding sites) after treating SCC/OSCC cells with VD_3_ or ED-71 (Harris *et al*. 1998; Rosli *et al*. 2013; Shintani *et al*. 2017). An in vivo experiment revealed that oral ED-71 administration significantly inhibited the growth of A431-derived tumors. The HBp17/FGFBP-1, FGF-2, Ki-67, and CD31 expression in the resected tumors of the ED-71-treated group was decreased compared with the vehicle control group. Thus, one of the effects of VD_3_ and ED-71 is to manipulate HBp17/FGFBP-1 expression in tumors, which in turn affects the release of FGF-2 from the ECM, which should inhibit angiogenesis (Fig. 1). In this experiment, no significant differences were observed in the gene and protein expression of CYP24A1, a metabolic enzyme that determines the biological half-life of ED-71 in tumors between the ED-71–treated and control groups. These results confirmed previous studies reporting the involvement of HBp17/FGFBP-1 in angiogenesis during tumor growth (Czubayko *et al*. 1997; Begum *et al*. 2007). These results indicate that ED-71 is particularly useful as a therapeutic agent for OSCC targeting the HBp17/FGFBP-1 molecule.

### ED71-induced microRNAs regulate HBp17/FGFBP-1

**Figure 2.**
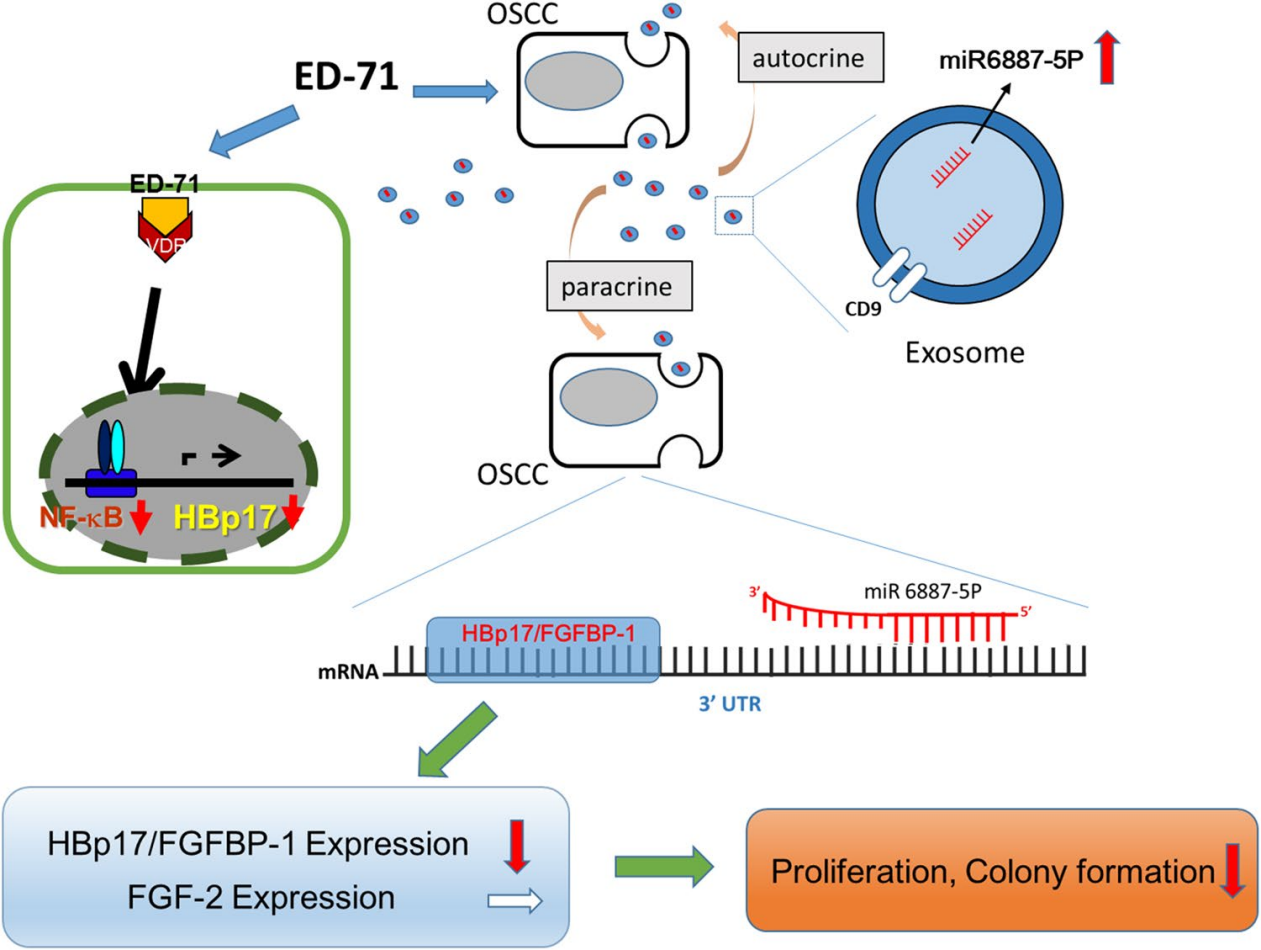
Schematic of the antitumor effect of exosomal miR-6887-5p–induced expression by ED-71 in SCC/OSCC cells. ED-71 induces the expression of exosomal miR-6887-5p secreted by SCC/OSCC cells, in addition to suppressing growth via the VDR-NFκB pathway. MiR-6887-5p suppressed the expression of HBp17/FGFBP-1 as a target by acting not only on secreted cells but also on surrounding cells in autocrine and paracrine manners, thereby suppressing cell proliferation and colony formation.

Growth factors, protein factors, hormones, lipids exosomes, and microRNAs (miRNAs) have been designated as regulatory chemical messengers (Sporn 2006). Exosomes act as messengers in intracellular communication networks through protein and RNA transfer between cells through both paracrine and autocrine mechanisms (Krämer-Albers and Hill 2016; Tkach and Théry 2016). Exosomes are potentially useful therapeutic biomarkers (Andaloussi *et al*. 2013; Pucci *et al*. 2016). Therefore, to search for miRNAs involved in HBp17/FGFBP-1 expression, we analyzed exosomal miRNAs from a medium conditioned by A431 cells treated with ED-71 in serum-free culture (Sato *et al*. 1987; Okamoto *et al*. 1996b). Microarray analysis revealed that 12 exosomal miRNAs were upregulated in ED-71–treated A431 cells compared with nontreated A431 cells (Higaki *et al*. 2020). MiR-6887-5p exhibited a predicted mRNA target matching the 3′-untranslated region (3′-UTR) of HBp17/FGFBP-1. A luciferase reporter assay revealed that the 3′-UTR of HBp17/FGFBP-1 was a direct target of miR-6887-5p in SCC/OSCC cells. The overexpression of miR-6887-5p in SCC/OSCC cells inhibited cell proliferation and colony formation in vitro and tumor growth in vivo compared with the control. We revealed a novel anticancer mechanism that involves HBp17/FGFBP-1 function regulation by exosomal miR-6887-5p in SCC/OSCC cells, which has potential as a target for miRNA-based cancer therapy (Fig. 2).

### Vitamin D_3_ supplementation

Recent randomized clinical trials revealed that VD_3_ supplementation prevented relapse in a subgroup of patients with digestive tract cancer who revealed adequate CD56-positive lymphocyte infiltration in the tumors (Akutsu *et al*. 2021). Their separate analysis revealed that vitamin D_3_ supplementation could increase and decrease serum programmed death-ligand 1 (PD-L1) levels when serum PD-L1 levels were low and high, respectively. VD_3_ supplementation significantly reduced the risk of mortality and recurrence or death by approximately one-third in the group with high serum PD-L1 levels compared with placebo (Morita *et al*. 2021). Statistically significantly higher infiltration of immune cells into the tumor and/or stroma was observed in patients with HNC with high 25-hydroxyvitamin D (25[OH]D) levels compared with those with low 25[OH]D levels, which was associated with longer overall survival (Bochen *et al*. 2018). However, the effect of VD_3_ supplementation on immune cells infiltrating SCC/OSCC remains to be elucidated (Ito *et al*. 2023). Therefore, not only does VD_3_ show an antitumor effect on SCC/OSCC cells, but it also affects the function of immune cells infiltrated into SCC/OSCC. Thus, vitamin D_3_ supplementation may be an effective adjunct treatment for SSC/OSCC in the future.

## Conclusion

This review described the results of our functional analysis of the HBp17/FGFBP-1 protein to determine its potential as a target for molecularly targeted therapy of SCC/OSCC with VD_3_. A recombinant HBp17/FGFBP-1 protein was exogenously added to SCC/OSCC cells, and the effect on cell proliferation ability was investigated; however, no significant difference was observed compared with the control. The HBp17/FGFBP-1 protein itself demonstrated no cell proliferation activity and is considered to serve as a pivot molecule that acts when FGF-2 is secreted outside of cells. The expression level of HBp17/FGFBP-1 in tumor cells demonstrated a correlation with cell proliferation, and HBp17/FGFBP-1 can be considered a therapeutic target molecule. Suppression of HBp17/FGFBP-1 expression in OSCC using vitamin D_3_ or eldecalcitol could be useful in the treatment of OSCC.

## Data Availability

Not applicable.
